# Perceived sources of stress among Malaysian dental students

**DOI:** 10.5116/ijme.5521.3b2d

**Published:** 2015-05-02

**Authors:** Muneer G. Babar, Syed S. Hasan, Yong J. Ooi, Syed I. Ahmed, Pei S. Wong, Siti F. Ahmad, Nik M. MNM-Rosdy, Normaliza A. Malik

**Affiliations:** 1 School of Dentistry, International Medical University (IMU), Kuala Lumpur, Malaysia; 2 School of Pharmacy, University of Queensland, Brisbane, Australia; 3 UG Student, School of Pharmacy, International Medical University; 4 School of Pharmacy, International Medical University (IMU), Kuala Lumpur, Malaysia; 5 Faculty of Dentistry, University of Malaya, Kuala Lumpur, Malaysia; 6 Faculty of Dentistry, Universiti Teknologi MARA (UiTM), Malaysia; 7 Faculty of Dentistry, University Science Islam Malaysia, Malaysia

**Keywords:** Dental students, stress level, stress sources, Malaysian universities

## Abstract

**Objectives:**

The study objectives were to identify the stress levels and to explore the impact of students' year of study and gender on the perceived sources of stress among Malaysian dental students.

**Methods:**

This was a cross-sectional study involving dental students from year one to year five from private and public universities in Malaysia. The study was formally approved by the Research and Ethics Committee, International Medical University Malaysia. Dental Environment Stress (DES) questionnaire was used for data collection and the gathered data were analyzed using SPSS® version 18. The Kruskal-Wallis and the Mann-Whitney U tests were used to compare stress items across various academic years and universities.

**Results:**

A total of five hundred and twenty nine (529) students participated in this study. Fear of failing the course at the end of year exams (mean stress level=5.57); concerns regarding completion of clinical work (mean=5.30); and examination results and grades (mean=5.27) were found as top stressors among dental students. Female students had higher stress scores than males with respect to personal issues, academic performance, educational environment and learning of clinical skills. Students from public universities had higher stress scores than their counterparts from private universities.

**Conclusion:**

The Malaysian dental students reported higher levels of stress. Present study identified stressors affecting dental students' academic life, and highlights the importance of stress management programs and other measures to minimize the impact of stress on both academic and personal lives of the students.

## Introduction

Stress is a physical or psychological phenomenon developed through self-cognition of provoking factors, after interacting with one's surroundings.^1^ Stress is experienced by many people in a variety of social, academic, and work settings. Although stress can be a source of motivation, excess stress can be debilitating.^2^

High levels of stress may have a negative impact on the students' learning ability. Studies have reported a high prevalence of stress among medical students ranging from 25–90%.^3^^,^^4^ A recently published systematic review reported that dental students also experience significant amounts of stress.^5^ Dentistry has been frequently rated as an exceptionally stressful profession; starting as a student and progressing into clinical practice after graduation.^6^^-^^8^ Naidu and colleagues reported that stress among first and second year dental students was mainly academic in comparison to largely clinical and interpersonal relationship-related stress among students during clinical years.^9^ This could be due to the pressures of starting clinical practice, financial problems, social issues with colleagues or patients, and the stress of practice management.^10^

Studies have shown that students who were under high stress continuously were emotionally exhausted and had been suffering from mental distress, physical manifestations, and eventually, burnout.^11^ Therefore studies have been done on dental students in different countries, focusing on stress during their learning and training stage.^2^^,^^7^^,^^8^ Interestingly some of the studies have also linked the perception of stress with gender, academic year and living environment.^12^ In 2009, Polychronopoulou et al suggested that dental undergraduates are constantly stressed as they need to be competent in both academic and clinical aspects as much as in interpersonal skills.^11^ They concluded that these perceived stress factors varied significantly between students from different institutions and were closely linked to gender, level of study, class size, type of curriculum and educational fees.^11^ Likewise Tangade et al reported fear of failure as the top stressor among the students and observed an increasing trend of stress from first through the final year of study.^13^ Between genders, studies in Europe, Japan and Saudi Arabia have shown that female students had higher stress scores than male students, whereas an Indian study found no difference.^11^^, ^^12^^-^^14^

In view of the literature, it is crucial to have a better understanding of students' perceived stress factors which would in turn contribute to building a positive and effective learning environment.^11^ Hence, the objective of the present study were to determine the stress levels and perceived sources and factors affecting stress among Malaysian dental students.

## Methods

### Study design and participants

This cross-sectional study was carried out among first to final year (5th year) dental students from one private and three public Malaysian universities. Students' participation was on a voluntary basis and their identities remained anonymous. The study was approved by the International Medical University (IMU) Joint Research and Ethics Committee. In addition, approval to conduct this study was obtained from the administration of each participating university.

### Questionnaire development and validation

The Dental Environment Stress (DES) questionnaire is a validated and commonly used tool, which has been previously used in many studies with similar objectives.^13^^-^^16^ The DES questionnaire is comprised of 40 questions relating to possible stressors and is divided into five sections (A-E), based on the 38-item DES stressors inventory introduced by Garbee et al.^16^ Part A identifies demographic information, whereas part B, C, D and E consist of 32 questions relating to stressors associated with different domains, i.e. Personal issues (PI), academic performance (AP), and educational environment (EE) and learning clinical skills (LCS). The questionnaire responses were based on a seven point Likert scale with 1 = not stressful at all, and 7 = very stressful.

The face and content validity of the DES was assessed by 10 academic staff from various disciplines and expertise. After addressing the feedback/comments received from the academicians the draft questionnaire was pre-tested on a sample of twenty students from dental school at IMU prior to its final use on study participants. The reliability of questionnaire was tested using Cronbach-alpha test and a value of 0.8 was considered reliable.^17^

### Sampling and data collection

A sample size calculation determined that 369 dental students would be necessary for this study. Due to differences in semester commencement dates and progress within the institutions, and in view of the difficulties in approaching students in the clinical years, a convenience sampling approach was adopted and a total of 529 students agreed to participate in this study. Therefore, we strongly believed that responses generated can be considered as a good representation of dental students' population in the country.

One faculty member from each participating university was briefed on the study aims, objectives, and methodology and had supervised the data collection at their respective institutions. However the primary researcher was available to clarify items and answer questions as needed. A self-administered questionnaire technique was used for the data collection. Prior to data collection students were briefed about the study objectives, in addition a study information sheet was provided and finally, written consents were obtained from the study participants. All gathered information was kept confidential.

### Statistical analysis

Statistical analysis was performed using SPSS® version 18. Descriptive statistics were analyzed using frequencies, percentages, mean, standard deviations and median. Chi-square and Spearman tests were used to determine the association and correlation of overall mean scores of each domain across genders, age groups, academic years, ethnicities and type of universities. Mean and standard deviation for each stressor were used to compare between different academic years, gender, and type of university. Kruskal-Wallis test and Mann Whitney U test were used to compare stress items between different academic years, and type of university. Previous studies have identified four main underlying factors. We used these factors as subgroups to investigate the differences between students from different years of study (first to final year) and type of university (private versus public).

## Results

### Socio-demographics of study population

A total of 529 dental students from four universities participated in this study, among them 404 (76.4%) were females while 125 (23.6%) were male students. Majority (68.6%) of the respondents were Malays followed by Chinese (28.5%) students, which is almost consistent with the Malaysian population ethnic distribution. The age range was 17 to 27 years with a mean age of 21 years. Dentistry was the first choice of study as reflected by the majority (87.9%) of the respondents. Table 1 provides the detailed socio-demographic characteristics of the study participants.

**Table 1:** Socio-demographics of the study population (n=529)

Variables		Overall n (%)	Public University n (%)	Private University n (%)
Gender				
	Male	125 (23.6)	96(76.8)	29 (23.2)
	Female	404 (76.4)	345 (85.40)	59 (14.60)
Age groups				
	17 - 20	225 (42.5)	191 (84.89)	34 (15.11)
	21-24	296 (56.0)	244 (82.43)	52 (17.57)
	25-27	8 (1.5)	6 (75)	2 (25)
Ethnic groups				
	Malay	363 (68.6)	360(99.17)	3 (0.83)
	Chinese	151 (28.5)	71 (47.02)	80 (52.98)
	Indian	7 (1.3)	4 (57.14)	3 (42.86)
	Others	8 (1.5)	6 (75)	2 (25)
Year of study				
	Year 1	124 (23.4)	96 (77.42)	28(22.58)
	Year 2	123 (23.2)	97 (78.86)	26 (21.14)
	Year 3	110 (20.8)	85 (77.27)	25 (22.73)
	Year 4	96 (18.1)	87 (90.63)	9 (9.37)
	Year 5	76 (14.4)	76 (100)	0 (0)

### Level of stress associated with personal issues

Female students showed a higher overall mean score for stress associated with personal issues compared to their male counterparts with significant differences observed in most of the items (Table 2). Interestingly year four students from private institution had the highest total mean score for personal issues compared to those from public universities highlighted a greater stress in students at private universities, however they had lower scores for fear of unemployment after graduation.

**Table 2.** Mean scores for personal issue, academic performance, educational environment, and learning clinical skills (n = 529)

Variables		Overall mean score for PI^*^	Overall mean score for AP^*^	Overall mean score for EE^*^	Overall mean score for LCS^*^	p-value^†^
Gender						
	Male	3.60	4.34	3.67	4.11	PI = 0.001; AP = 0.001; EE = 0.001; LCS = 0.001
	Female	3.95	4.95	3.95	4.60	
Age groups						
	17 - 20	3.84	4.80	3.49	3.98	PI = 0.001; AP = 0.001; EE = 0.001; LCS = 0.001
	21-24	3.88	4.80	4.18	4.85	
	25-27	3.85	5.06	3.96	4.90	
Ethnic groups						
	Malay	3.97	4.96	4.00	4.58	PI = 0.001; AP = 0.001; EE = 0.001; LCS = 0.001
	Chinese	3.61	4.43	3.65	4.27	
	Indian	3.36	4.61	3.12	3.97	
	Others	4.14	4.77	3.38	4.22	
Year of study						
	Year 1	3.78	4.79	3.17	3.49	PI = 0.001; AP = 0.001; EE = 0.001; LCS = 0.001;
	Year 2	3.86	4.77	3.78	4.49	
	Year 3	3.79	4.51	3.84	4.63	
	Year 4	4.00	4.89	4.22	4.96	
	Year 5	3.92	5.18	4.84	5.25	
Type of university						
	Public	3.92	4.89	3.97	4.61	PI = 0.001; AP = 0.001; EE = 0.001; LCS = 0.001
	Private	3.59	4.37	3.42	3.82	

*PI = personal issue, AP = academic performance, EE = education environment, LCS = learning clinical skills. †Association assessed by Chi-square test.

### Level of stress associated with academic performance

Fear of failing a course, an academic year, examination and grades were rated as top stressors in this category. However, females and year five students appeared to be more stressful due to these academic performance related stressors. Interestingly again, students from public universities had higher stress level than their counterparts at private institutions and the difference observed was statistically significant except for examination and grades (Table 3).

**Table 3:** Differences in stress levels associated with academic performance, educational environment and learning clinical skills from type of university perspective (n=529)

Questions	Type of university^*^	Mean score	Difference p-value
Amount of assigned work	1	4.77	0.001
	2	4.24	
Competition with peers for grades	1	4.71	0.001
	2	4.11	
Lack of time to do assigned work	1	5.01	0.017
	2	4.55	
Inapproachability of the teaching staff	1	4.05	0.001
	2	3.24	
Fear of failing a course or the year	1	5.64	0.029
	2	5.13	
Lack of time for reflective learning	1	4.78	0.001
	2	4.06	
Stress levels with educational environment			
Lack of cooperation by lecturers and patients	1	4.18	0.001
	2	3.24	
Criticism on work by the faculty members	1	4.02	0.001
	2	3.43	
Inconsistency of feedback on your work by faculty members	1	4.00	0.046
	2	3.72	
Rules and regulation of the faculty	1	3.92	0.001
	2	3.30	
Stress levels with learning clinical skills			
Lecturers or patients being late or not showing for their appointments	1	4.63	0.001
	2	3.50	
Difficulty in learning clinical skills	1	4.47	0.014
	2	4.08	
Atmosphere created by clinical supervisors	1	4.36	0.001
	2	3.47	
Receiving criticism from staff for clinical work	1	4.44	0.001
	2	3.47	
Difficulty in learning and interpreting laboratory findings	1	4.32	0.002
	2	3.85	
Completion of clinical requirements	1	5.37	0.001
	2	4.08	
Shortage of allocated clinical time	1	5.22	0.001
	2	4.17	

*1=public, 2=private, only statistically significant items are included

### Level of stress associated with education environment

There was an increasing trend in mean stress scores from year one to year five (Table 2). Year five students were significantly more stressed than year-one students in all items and similar to previous category, females reported more stress than males students. As for the attitude of faculties, no significant differences were seen between the perceptions of public and private universities students. No significant differences were detected in students' perception of attitudes of faculty towards students between the universities (Table 3).

### Level of stress associated with learning clinical skills

Among the eight stressors listed in this domain, completion of clinical requirements was the most stressful item for students in the clinical phase, followed by shortage of allocated clinical time (Table 4). Again, female students reported more stress than male counterparts in learning clinical skills. Overall students from public universities perceived greater stress than students in private university (Table 3).

**Table 4:** Differences in Level of stress associated with learning clinical skills of students from different professional years* (n=529)

Questions	Year of study	Mean scores	Overall difference p-value
Responsibilities for comprehensive patient care	1	3.23	0.001
	2	4.21	
	3	4.35	
	4	4.24	
	5	4.51	
Lecturers or patients being late or not showing for their appointments	1	3.03	0.001
	2	4.16	
	3	4.65	
	4	5.25	
	5	5.87	
Difficulty in learning clinical skills	1	3.71	0.001
	2	4.67	
	3	4.45	
	4	4.66	
	5	4.72	
Atmosphere created by clinical supervisors	1	3.35	0.001
	2	4.18	
	3	4.22	
	4	4.59	
	5	5.18	
Receiving criticism from staff for clinical work	1	3.51	0.001
	2	4.37	
	3	4.52	
	4	4.56	
	5	4.70	
Difficulty in learning and interpreting laboratory findings	1	3.75	0.042
	2	4.33	
	3	4.29	
	4	4.48	
	5	4.51	
Shortage of allocated clinical time	1	3.64	0.001
	2	5.08	
	3	5.26	
	4	5.70	
	5	6.12	

* Only statistically significant items are included

## Discussion

The findings of this study provided an interesting and useful insight into a subject that is often not viewed as a priority. The top stressors perceived by dental students are related to academic issues, which is consistent with previous investigations.^6^^,^^13^ Fear of failure in a course was ranked as the most stressful item across all professional years and appeared similar to what was reported by Tangade and Colleagues.^13^ These findings may help in improving strategize to which may enable students to overcome the academic life related stressors. Similarly, while viewing various aspects of academic stress, it was also found that senior (final year) students expressed greatest stress in all the academic related items, which might be an indication of the pressure to graduate while facing the challenges of professional life. Year three students in the present study had the lowest overall academic stress, which again found in concordance with other reported studies.^13,17 ^However, in contrast to previous studies, we found peak in stress level among third year students which coincides with the transition from pre-clinical to clinical phase.^9^^,^^13^^,^^19^^,^^20^ Stressed students may become detached and unable to focus their emotions and intentions, therefore it is worth noting that an earlier Malaysian study had found that third-year dental students had the lowest mean empathy score when compared to students in other study years.^21^

In the context of stress due to personal issues, students from clinical years reported significantly higher anxiety, related to lack of confidence in becoming a successful professional and fear of being unable to catch up if getting behind in the studies. This may be due to their busy schedule combined with their aspiration to be competent both academically and clinically. Another interesting finding was that fear of unemployment upon graduation was perceived most stressful among students in their initial years with a decreasing trend seen as they proceed to the clinical years. Studies in India and Nigeria found the opposite trend and students were more anxious about their future as they progressed.^11^^,^^19^ Greater job security in Malaysia caused by paid compulsory government service and vacancy in private practice may explain the relatively lower fear of unemployment.

Increasing trends in stress were observed in both educational environment and learning clinical skill domains, where lack of cooperation from lecturers and patients raised concern among students. In addition students worried about inconsistent feedback from their lecturers. Peretz et al have also reported inconsistency in receiving feedback as a major source of stress.^23^ One interpretation is that senior students' reported greater stress in education and clinical skills domains with increased interaction with faculty members and patients as well as high expectation from faculty members as they enter the clinical phase. This interpretation is consistent with findings from Greece and India.^19^^-^^20^ Our findings that senior years student suffered from greater stress are supported by previous studies suggesting that the coursework becomes more difficult with each passing year.^9^^,^^12^^,^^24^ Noteworthy differences were observed when items were analyzed across gender. Female students reported higher levels of stress than their male counterpart in all aspects, which is in agreement with previous studies.^6^^,^^14^^,^^25^^,^^26^ However, Sofola et al reported that stress levels were not different between genders while Archarya et al reported that male students perceived greater overall stress.^20^^,^^21^ Higher stress reported by females may be due to the way they respond to stressful events and males being less expressive of their worries.^26^^,^^12^ From our results, it can be projected that almost one quarter (24.2%) of the students opted for the dental programme due to parental pressure. Parents who micromanage their kids in every decision "helicopter parents" may also lead to academic stress and this may have long term detrimental effects on their academic and professional life.

Finally, students from public institutions appeared more stressed than their private university counterparts. This may be due to differences in English language requirements and assessment (type and frequency) of private versus public universities. Surprisingly, no significant differences related to financial resources were found even though tuition fee at private institutions can be very high. This might due to the availability of educational loans by the government as well as scholarships and education sponsored funds by private companies. Furthermore, students who registered for dental courses in private universities in Malaysia may be able to afford the education costs and thus express lesser financial pressure. As expected, fear of unemployment upon graduation was the only stressor where students from private universities scored higher, perhaps due to the worries of getting high income jobs to repay on their investment.

### Limitations

The study involved students mainly from public institutions (441 students, 83.3%) and hence it may not clearly reflect the stress related factors of students in private institutions (88 students, 16.7%), though we may have some representation of it. Similarly, more female students participated in this survey than males (404 female vs. 125 male), therefore male responses may not be generaliseable.

## Conclusions

The students reported higher levels of stress. The most frequently occurring stressors among the students were are related to academic issues. Academic and non-academic stressors should be considered in curriculum planning and a mechanism should be in place to monitor and address dental students' stress. Stressed students may benefit by improving academic support systems via senior students, academic staff and counselor driven continuous mentoring. To accomplish this requires increased stress awareness, improved coping skills and focused support. In some cases involvement of the parents may be necessary. The experience of personal and professional stress does not end at graduation, students must be equipped with the necessary skills to assess personal distress, determine its effect on professional growth, recognize when to seek help, and develop strategies to promote personal well-being.

### Implications and future directions

A longitudinal study involving a cohort of students over five years academic periods can help provide deeper insight into real life stresses affecting students. Studies using qualitative methods are recommended for a better understanding of the sources of stress and how they may be managed best, based on the views of the stakeholders. Interventional studies should also be designed to measure the effectiveness of existing support system in place.

### Acknowledgements

We wish to record our appreciation to the participating institutions in this study.

### Conflict of Interest

The authors declare that they have no conflict of interest.
